# Study of the soliton propagation of the fractional nonlinear type evolution equation through a novel technique

**DOI:** 10.1371/journal.pone.0285178

**Published:** 2023-05-22

**Authors:** U. H. M. Zaman, Mohammad Asif Arefin, M. Ali Akbar, M. Hafiz Uddin

**Affiliations:** 1 Department of Mathematics, Jashore University of Science and Technology, Jashore, Bangladesh; 2 Department of Applied Mathematics, University of Rajshahi, Rajshahi, Bangladesh; China University of Mining and Technology, CHINA

## Abstract

Nonlinear fractional partial differential equations are highly applicable for representing a wide variety of features in engineering and research, such as shallow-water, oceanography, fluid dynamics, acoustics, plasma physics, optical fiber system, turbulence, nonlinear biological systems, and control theory. In this research, we chose to construct some new closed form solutions of traveling wave of fractional order nonlinear coupled type Boussinesq–Burger (BB) and coupled type Boussinesq equations. In beachside ocean and coastal engineering, the suggested equations are frequently used to explain the spread of shallow-water waves, depict the propagation of waves through dissipative and nonlinear media, and appears during the investigation of the flow of fluid within a dynamic system. The subsidiary extended tanh-function technique for the suggested equations is solved for achieve new results by conformable derivatives. The fractional order differential transform was used to simplify the solution process by converting fractional differential equations to ordinary type differential equations by using the mentioned method. Using this technique, some applicable wave forms of solitons like bell type, kink type, singular kink, multiple kink, periodic wave, and many other types solution were accomplished, and we express our achieve solutions by 3D, contour, list point, and vector plots by using mathematical software such as MATHEMATICA to express the physical sketch much more clearly. Moreover, we assured that the suggested technique is more reliable, pragmatic, and dependable, that also explore more general exact solutions of close form traveling waves.

## 1. Commencement & foreword

Fractional calculus (FC) is considered an influential instrument for more precisely describing real-world issues than differential calculus (DC). FC dates to the 1600s, when G.W. Leibnitz asked L’Hospital if an integer-order derivative could be applied to non-integer derivatives [1]. In engineering and mathematics, it would be a valuable method for understanding complex systems. It is a broader version of traditional order integration and differentiation. The usefulness of explicit solutions of the traveling wave for the non-linear fractional order partial type differential equations (NLFPDEs) is notable in the present condition [2]. Recently, NLFPDEs have become popular in optical fiber, geochemistry, chemical physics, fractional dynamics, biophysics, biomechanics, chemical kinematics, relativistic, fluid mechanics, gas dynamics, signal transmission, plasma physics, control theory, earthquakes, solid-state physics, ecosystem, and many other. Fractional order models work on a variety of scales, including the nanoscale, mesoscale, microscale, and macroscale. They are also used in social sciences like dietary supplements, environment, money, and financial concerns. Many additional types of fractional nonlinear evolution equations (FNLEEs) have been used to extract velocity wave propagation, which has piqued the scientific community of curiosity. It is used to simulate wave circulation and heat transfer in physics, as well as numerous types of population models to the atmospheric engineering [3–7].

There are many effective techniques available, like the Hirota-bilinear method [8,9], the exp-function approach [10], the first-integral technique [11], the $\bar{\partial }$-dressing method [12], the Riemann-Hilbert approach [13,14], the generalized exponential rational function approach [15], the Ansatz and sub equation theories [16], the (*G*′/*G*)- expansion approach [17], the Jacobi elliptic function technique [18], the modified auxiliary expansion technique [19], Lie symmetry analysis method [20],direct algebraic approach [21], the $\bar{\partial }$-stepest descent method [22,23], improved Bernoulli sub-equation Function technique [24], the Sine-Gordon expansion method [25] and residual power series technique [26], which effectively established for solving the solution to NLFPDEs.

The extended tanh-function approach is the most extensively used of them. By establishing tanh as a new variable for such a dependable treatment of nonlinear wave equations, Malfiet [27] pioneered the introduction of the practical and popular tanh-function approach. Tanh’s scheme was developed to find the solutions of traveling wave. Wazwaz [28] presented the expanded tanh technique afterward. In addition, the equations that can be solved using the extended tanh technique have a unique set of solutions known as solitons. Solitons are special traveling waves that maintain their shape even when colliding with others. From hydrodynamics to nonlinear optics, tsunamis to turbulence, plasmas to shock waves, traffic flow to the internet, and tornados to jupiter’s great red spot, this property is applicable in a wide variety of situations. Solitons have gained a great deal of interest recently in quantum fields and nano-technology, particularly in nano-hydrodynamics [29].

Space-time fractional order coupled type Boussinesq–Burger (BB) and Boussinesq equation are a prominent nonlinear type partial differential equation in this framework.

Let the space-time fractional order coupled BB equation,

$$D_{t}^{\alpha }U-\frac{1}{2}V_{x}+2UU_{x}=0$$ (1A)

$$D_{t}^{\alpha }V-\frac{1}{2}U_{xxx}+2{\left(UV\right)}_{x}=0\;0<\alpha <1,t>0,$$ (1B)

The space-time fractional order coupled Boussinesq equation is under consideration,

$$U_{x}+V_{x}=0$$ (2A)

$$D_{t}^{\alpha }V+\lambda {\left(u^{2}\right)}_{x}-\nu D_{xxx}^{3\alpha }U=0\;0<\alpha <1,t>0,$$ (2B)

The field of horizontal velocity is denoted by U(x,t) andV(x,t) represents the altitude of the water’s surface above the bottom-most horizontal level for the above two equations.

These two equations are a fascinating equational model of mathematics which illustrates the transmission of shallow water waves and emerges throughout the study of fluid flow inside a dynamical system. The shallow water equation is often a set of partial differentials (PD) equations which define the fluid flow underneath a surface pressure, flow within vertically well-mixed water bodies, and water body motion. Coastal engineers benefit greatly from a thorough understanding of their solutions to building harbors and coastlines using nonlinear water wave theory [30].

Various approaches were applied to explain the space-time fractional order coupled type BB equation for example M. H. Heydari and Z. Avazzadeh were solving this equation using the Orthonormal Bernoulli polynomials (OBPs) with derivative in the Caputo form [31], Kumar et. al. [32] developed this equation with Caputo derivative by Residual power series, Homotopy analysis transform method, and Homotopy polynomials and Decomposition technique by Caputo derivatives were used to explain the suggested equation by Mahmoud S Alrawashdeh and Shifaa Bani-Issa [33]. Alternatively, the space-time fractional order coupled type Boussinesq equation was solved by modified extended tanh technique with the modified Riemann-Liouville derivative developed the Khalid K. Ali et al. [34] and Handan C¸erdik Yaslan and Ayse Girgin established this proposed equation using Exp-function method with conformable derivative [10].

The core objective of the study is to identify innovative exact solutions of the NLFPDEs such as solitons, bells, kinks, and some other kinds of solutions. Those mentioned equations were solved using the extended tanh-function approach. Therefore, solving those proposed equations using the mentioned method with conformable derivatives is completely new. This strategy is used in this research to acquire more current and wide solutions that are easy to implement, configurable, expandable, faster to simulate, and flexible. The solutions are represented in terms of 3D and contour patern additionally with new graphical styles, like vector plot and list point plot, Applying those four sorts of pictographic descriptions, the physical phenomena of our suggested models define more clearly.

The remains of the paper will be rearranging in succeeding manner: In Section 2, the definition and base configuration were presented. Section 3, the execution of methodology for the extended hyperbolic tangent technique has been introduce. Also, in Section 4, we applied previously suggested technique to solving the specific answer for the mentioned equation. Additionally, Section 5 depicted a brief discussion, along with a graphical description and classification, that are given the photographic clarification. And the last section is standing the conclusion.

## 2 Definition and base configuration

Khalil et al. [35] have newly suggested the conformable derivative. Take up the function *f*:[0, ∞)→ℝ and the *α*-order “conformable derivative” of *f*, that is determine as:

$$T_{\alpha }(f)(t)=\underset{\varepsilon \to 0}{\lim}\frac{f(t+\varepsilon t^{1-\alpha })-f(t)}{\varepsilon },$$ (2.1)

for all *t*>0, *α*∈(0,1). If *f* is *α*-differentiable for some(0,a), a>0, and $\underset{t\to 0^{+}}{\lim}f^{\left(\alpha \right)}(t)$ seems to be, then to explain $f^{\left(\alpha \right)}(0)=\underset{t\to 0^{+}}{\lim}f^{\left(\alpha \right)}(t)$. The following formulas expresses how the use of conformable derivatives to demonstrate a few assumptions.

For the derivatives of quantitative measurements, addition, and products of the differentiable functions, the conformable derivative on time scales is essential, and this is accomplished to using the aforementioned formula [36]. Consistent with the definition supplied by Khalil et al.[35], the theorem given bellow satisfies with conformable derivative, which gives several important qualities.

### Theorem 1

Taking *α*∈(0, 1] and let, *f*, *g* were *α*-differentiable at all point *t*>0.

Additionally

- $T_{\alpha }(cf+dg)=cT_{\alpha }(f)+dT_{\alpha }(g)$, for all c,d∈R.
- $T_{\alpha }(t^{p})=pt^{p-\alpha }$, for all p∈R.
- $T_{\alpha }(c)=0$, for all constant function f(t)=c.
- $T_{\alpha }(fg)=fT_{\alpha }(g)+gT_{\alpha }(f)$.
- $T_{\alpha }(\frac{f}{g})=\frac{gT_{\alpha }(f)-fT_{\alpha }(g)}{g^{2}}$.
- So, if *f* transforms to differentiable, and then $T_{\alpha }(f)(t)=t^{1-\alpha }\frac{df}{dt}$.

Regarding the conformable derivative, Khalil [35] defines several more properties, like the inequality of Gronwall’s, several integration methods, exponential function, chain law, transformation of Laplace, and expansion of Tailor series.

#### Theorem 2

Let that an *α*-differentiable formulation was *f* in conformable differentiable as well assume that *g* develop as well as differentiable and determine the interval of *f*. And at that time

$$T_{\alpha }(f\circ g)(t)=t^{1-\alpha }g^{\prime }(t)f_{g}(t).$$

### 3 Execution of the methodology

Malfiet [27] developed the efficient tanh approach for a reliable resolution of nonlinear wave equations in one of his pertinent features. Following that, Wazwaz [28] improved the extended tanh method. The key idea behind the suggested approach is to represent a polynomial solution in the form of hyperbolic type functions, and then to characterize the variable coefficient of PDE by resolving a combination of arithmetical equations and first-order ODEs.

To initiate, we apprehend a particular nonlinear equation PDE U(x,t) is defined by

$$P(U,U_{x},U_{t},U_{xt},U_{xx},U_{tt},\ldots )=0.$$ (3.1)

In this example, there x and t are two independent variables.

The fundamental NLFPEE construction is assumed to be follows:

$$P(U,D_{t}^{\alpha }U,D_{x}^{\beta }U{,D}_{t}^{\alpha }D_{t}^{\alpha }U,D_{t}^{\alpha }D_{x}^{\beta }U,D_{x}^{\beta }D_{x}^{\beta }U,\ldots \;\ldots \;\ldots )=0,0<\alpha ,\beta \leq 1$$ (3.2)

Wherever *α*, *β* are fractional order derivatives,U symbolizes a function of t and x are the temporal derivative and spatial derivative correspondingly, then polynomial of U(x,t) is symbolizes by *P* and its derivatives where supreme order of linear derivatives also the maximum order nonlinear derivatives are allied. Consider the transformation of waves.


$$\zeta =k\frac{x^{\beta }}{\beta }+c\frac{t^{\alpha }}{\alpha },U(x,t)=U(\zeta ),$$ (3.3)

Here k and c are independent constants with nonzero values.

If we wanted to use this wave transformation in (3.2), we should rewrite it like this:

$$Q(U,U^{\prime },U^{\prime \prime },U^{\prime \prime \prime },\ldots \;\ldots \;\ldots )=0$$ (3.4)

The Eq (3.4) represents U as the ordinary derivative.

#### Phase 1

Let us take a formal solution of ODE (3.4) to the following framework

$$U(\zeta )=\sum_{i=0}^{n}a_{i}Y^{i}+\sum_{i=1}^{n}b_{i}Y^{-i},$$ (3.5)

along with

Y=tanh(μζ), (3.6)

where *a*_*i*_, *b*_*i*_, *μ* is any arbitrary constant which are to be determine afterword’s.

#### Phase 2

Let the homogeneous balance to the derivatives of the linear terms which is maximum order and nonlinear terms which is maximum order appearing in Eq (3.4) to presume *n* be a constant, which is positive.

#### Phase 3

Substitute solution (3.5) with (3.6) to Eq (3.4) through the rate of *b*_*i*_ derived into phase 2, and we have got polynomials *Q*. Organizing every coefficient to the corresponding polynomials to zero provides a collection of arithmetical equations for $a_{i}^{\prime }s$ and $b_{i}^{\prime }s$. Obtain the following series of equations for $a_{i}^{\prime }s$ and $b_{i}^{\prime }s$ by virtue of the figurative computer software, as, for example, Maple.

#### Phase 4

Implanting values occurred in phase 3 to Eq (3.5) in the company of Eq (3.6), we generate closed form solutions of traveling wave for nonlinear evolution Eq (3.2).

## 4 Analyzing causes and proposing solutions

By applying the mentioned technique, we try to find more accurate exact analytic solutions of wave for the essential FNLEEs, that is, the space-time fractional coupled BB and coupled Boussinesq equations via conformable derivative concept.

### 4.1 The space-time fractional coupled Boussinesq–Burger (BB)

We already introduced the space-time fractional order coupled BB equation by Eqs (1A) and (1B). Then, by applying the consequent nonlinear complex transformation of wave:

$$\zeta =x-c\frac{t^{\alpha }}{\alpha },\;U(x,t)=U(\zeta ),$$ (4.1.1)

where *c* denoted the speed of traveling wave. Put on Eq (4.1.1), to Eqs (1A) and (1B) reduces to integration of ODE:

$$-cU^{\prime }-\frac{1}{2}V^{\prime }+2UU^{\prime }=0$$ (4.1.2A)

$$-cV^{\prime }-\frac{1}{2}U^{\prime \prime \prime }+2{\left(UV\right)}^{\prime }=0$$ (4.1.2B)

where the derivative of *ζ* is symbolizes by ′ regarding U. Integrating the Eqs (4.1.2A) then (4.1.2B) with respect to *ζ* one time and considering the integrating zero constant and we find

$$-cU-\frac{1}{2}V+U^{2}=0$$ (4.1.3A)

$$-cV-\frac{1}{2}U^{\prime \prime }+2UV=0.$$ (4.1.3B)

Equation leads to the following conclusion (4.1.3A):

$$V=2(U^{2}-cU).$$ (4.1.4)

Eq (4.1.4) is replaced to Eq of (4.1.3B) and we have got,

$$-\frac{1}{2}U^{\prime \prime }+4U^{3}+2c^{2}U-6cU^{2}=0.$$ (4.1.5)

Balancing to the maximum order non-linear term $U^{3}$ and the linear term $U^{\prime \prime }$ of maximum order produces the homogeneous balance, which was one. So, the result of Eq (4.1.5) taking the form bellow:

$$U(\zeta )=a_{0}+a_{1}Y+b_{1}Y^{-1},$$ (4.1.6)

where the evaluated constants are *a*_0_, *a*_1_ *and b*_1_. The left-hand side turns out be a polynomial in *Y* by replacing (4.1.6) to (4.1.5) with (3.6). Tapping a null value in each of this polynomial’s coefficients produces a set of arithmetical equations of *a*_0_, *a*_1_, *b*_1_ and c (which, for the sake of clarity, we do not display.). In this situation, we had to suppose the particular values, which are $\mu =c=1\;and\;\alpha =\frac{1}{2}$ and we apply the Maple software to estimate those values. Also, we resolve a generalized set of equations using those values, as illustrated in the results below:

## Cluster 1


$$c=\mu ,a_{0}=\frac{1}{2}\mu ,a_{1}=\frac{1}{2}\mu \;and\;b_{1}=0.$$

The performance parameters in Cluster 1 afford an explicit solution for *tanh* functions, and it is transformable into another form using the hyperbolic formula and coordinates in space and time (4.1.1).


$$U_{1_{1}}(x,t)=\frac{1}{2}+\frac{1}{2}\tanh\left(x-2\sqrt{t}\right).$$ (4.1.7A)

$$V_{1_{1}}(x,t)=2[{\left\{\frac{1}{2}+\frac{1}{2}\tanh\left(x-2\sqrt{t}\right)\right\}}^{2}-\{\frac{1}{2}+\frac{1}{2}\tanh\left(x-2\sqrt{t}\right)\}].$$ (4.1.7B)

## Cluster 2


$$c=\mu ,a_{0}=\frac{1}{2}\mu ,a_{1}=-\frac{1}{2}\mu \;and\;b_{1}=0.$$

The given parameters in Cluster 2 deliver the solution for *tanh* and *coth* functions, which can be adjusted with the aid of hyperbolic formulation and space and time coordinates, using transformation of (4.1.1).

## Cluster 3


$$c=2\mu ,a_{0}=\mu ,a_{1}=\frac{1}{2}\mu \;and\;b_{1}=\frac{1}{2}\mu .$$

For *tanh* and *coth* functions, Cluster 3’s parameters afford an explicit solution, and it is transformable into another form by means of the hyperbolic formula and coordinates in space and time (4.1.1).


$$U_{1_{3}}(x,t)=1+\frac{1}{2}tanh(x-4\sqrt{t})+\frac{1}{2}coth(x-4\sqrt{t})$$ (4.1.9A)

$$V_{1_{3}}(x,t)=2[\{{1+\frac{1}{2}tanh(x-4\sqrt{t}]+\frac{1}{2}coth(x-4\sqrt{t})\}}^{2}-\{1+\frac{1}{2}\tanh\left(x-4\sqrt{t}\right)+\frac{1}{2}\coth\left(x-4\sqrt{t}\right)\}].$$ (4.1.9B)

## Cluster 4


$$c=-2\mu ,a_{0}=-\mu ,a_{1}=\frac{1}{2}\mu \;and\;b_{1}=\frac{1}{2}\mu .$$

For *tanh* and *coth* functions, Cluster 4’s parameters offer an explicit solution that may be changed by satisfying hyperbolic formulation, time and space coordinates, and transformation of (4.1.1).


$$U_{1_{4}}(x,t)=-1+\frac{1}{2}tanh(x+4\sqrt{t})+\frac{1}{2}coth(x+4\sqrt{t})$$ (4.1.10A)

$$V_{1_{4}}(x,t)=2[\{{-1+\frac{1}{2}\tanh\left(x+4\sqrt{t}\right)+\frac{1}{2}\coth\left(x+4\sqrt{t}\right)\}}^{2}-\{-1+\frac{1}{2}\tanh\left(x+4\sqrt{t}\right)+\frac{1}{2}Coth(x+4\sqrt{t})\}].$$ (4.1.10B)

## Cluster 5


$$c=2\mu ,a_{0}=\mu ,a_{1}=-\frac{1}{2}\mu \;and\;b_{1}=-\frac{1}{2}\mu$$

The parameters of Cluster 5 provide an explicit solution for *tanh* and *coth* functions, this can be modified using the hyperbolic formula and the translation (4.1.1) of time and space.


$$U_{1_{5}}(x,t)=1-\frac{1}{2}tanh(x-4\sqrt{t})-\frac{1}{2}coth(x-4\sqrt{t})$$ (4.1.11A)

$$V_{1_{5}}(x,t)=2[\{{1-\frac{1}{2}\tanh\left(x-4\sqrt{t}\right)-\frac{1}{2}\coth\left(x-4\sqrt{t}\right)\}}^{2}-\{1-\frac{1}{2}\tanh\left(x-4\sqrt{t}\right)-\frac{1}{2}\coth\left(x-4\sqrt{t}\right)\}].$$ (4.1.11B)

## Cluster 6


$$c=\mu ,a_{0}=\frac{1}{2}\mu ,a_{1}=0\;and\;b_{1}=\frac{1}{2}\mu$$

An explicit solution for the *tanh* and *coth* functions is involved in merge 6’s yield parameters, this can be changed by applying the transformation (4.1.1) to time and space coordinates and the hyperbolic formula.


$$U_{1_{6}}(x,t)=\frac{1}{2}+\frac{1}{2}coth(x-2\sqrt{t})$$ (4.1.12A)

$$V_{1_{6}}(x,t)=2[\{{\frac{1}{2}+\frac{1}{2}coth(x-2\sqrt{t})\}}^{2}-\{\frac{1}{2}+\frac{1}{2}\coth\left(x-2\sqrt{t}\right)\}].$$ (4.1.12B)

## Cluster 7


$$c=i\sqrt{2}\mu ,a_{0}=\frac{i}{\sqrt{2}}\mu ,a_{1}=-\frac{1}{2}\mu \;and\;b_{1}=\frac{1}{2}\mu .$$

Cluster 7 yield parameters include an explicit solution to the tanh functions, this can be changed by applying the transformation (4.1.1) to time and space coordinates and the hyperbolic formula.


$$U_{1_{7}}(x,t)=\frac{i}{\sqrt{2}}-\frac{1}{2}\{1-\mathrm{sech}\left(x-i2\sqrt{2}\sqrt{t}\right)\}+\frac{1}{2}coth(x-i2\sqrt{2}\sqrt{t})$$ (4.1.13A)

$$V_{1_{7}}(x,t)=2[[{\frac{i}{\sqrt{2}}-\frac{1}{2}\{1-\mathrm{sech}\left(x-i2\sqrt{2}\sqrt{t}\right)\}+\frac{1}{2}coth(x-i2\sqrt{2}\sqrt{t})]}^{2}-[\frac{i}{\sqrt{2}}-\frac{1}{2}\{1-\mathrm{sech}\left(x-i2\sqrt{2}\sqrt{t}\right)\}+\frac{1}{2}\coth\left(x-i2\sqrt{2}\sqrt{t}\right)].$$ (4.1.13B)

It really should be presumed that the solution $U_{1_{1}}-U_{1_{7}}$ and $V_{1_{1}}-V_{1_{7}}$ of the space-time fractional order coupled BB equation is fresh and this work was the one who eventually detected it, which was mysterious and unexplored in previous works.

### The space-time fractional coupled Boussinesq equation

By using Eqs (2A) and (2B), we have previously presented space-time fractional order coupled type Boussinesq equation. Afterwards, we employ simultaneous nonlinear complex transformation of wave:

$$\zeta =k\frac{x^{\alpha }}{\alpha }-c\frac{t^{\alpha }}{\alpha },U(x,t)=U(\zeta ),$$ (4.2.1)

where *c* denote the speed of traveling wave. Put on Eq (4.2.1), to Eqs (2A) and (2B) diminish to the next integral order ODE:

$$-cU^{\prime }+kV^{\prime }=0$$ (4.2.2A)

$$-cV^{\prime }+\lambda {\left(U^{2}\right)}^{\prime }-\nu k^{3}U^{\prime \prime \prime }=0,$$ (4.2.2B)

where $\prime =\frac{d}{d\zeta }$. Integrating the Eq (4.2.2A) and the Eq (4.2.2B) with respect to *ζ* one time and considering the integrating constant is zero and we find

−cU+kV=0 (4.2.3A)

$$-cV+\lambda U^{2}-\nu k^{3}U^{\prime \prime }=0.$$ (4.2.3B)

From Eq (4.2.3A), we may infer the following:

$$V=\frac{c}{k}U.$$ (4.2.4)

Eq (4.1.4) is substituted into the Eq of (4.1.3B), yields

$$-\nu k^{4}U^{\prime \prime }+\lambda kU^{2}-c^{2}U=0.$$ (4.2.5)

Taking the balance to the maximum order $U^{2}$ of nonlinear term and the linear term $U^{\prime \prime }$ of highest order, yields the homogeneous balance is two. Thus, the result of Eq (4.2.5) is taking the form bellow:

$$U(\zeta )=a_{0}+a_{1}Y+a_{2}Y^{2}+b_{1}Y^{-1}+b_{2}Y^{-2},$$ (4.2.6)

Where the evaluated constants are *a*_0_, *a*_1_, *a*_2_, *b*_1_ and *b*_2_. The left-hand side turns to be a polynomial in Y by replacing (4.2.6) to (4.2.5) through (3.6). Putting a null value in each of this polynomial’s coefficients produces a set of arithmetical equations for *a*_0_, *a*_1_, *a*_2_, *b*_1_, *b*_2_, kandc (that we neglect to display for clarity). Here we use software maple to estimate those values, at that time consider the values are $\mu =\nu =k=c=1\;and\;\alpha =\frac{1}{2}.$ So we get V=U by means of those values. Using those values, we explain a generalized group of equations, for example exposed in the table below:

## Assemble 1


$$c=2\sqrt{\nu }k^{2}\mu ,k=k,a_{0}=-\frac{2\nu k^{3}\mu^{2}}{\lambda },a_{1}=0,a_{2}=0,\;b_{1}=0,\;and\;b_{2}=\frac{6\nu k^{3}\mu^{2}}{\lambda }.$$

Assemble 1’s elements influencing the *coth* functions deliver an explicit solution by the transformation of (4.2.1), time-space coordination, and hyperbolic formula, we get,

$$U_{2_{1}}=-2+6{coth(2\sqrt{x}-4\sqrt{t})}^{2}.$$ (4.2.7)

The results (4.2.7) can be written as follows using the trigonometric formula:

$$U_{2_{2}}=-2+6\{1+{\mathrm{csch}\left(2\sqrt{x}-4\sqrt{t}\right)}^{2}\}.$$ (4.2.8)

## Assemble 2


$$c=2\sqrt{\nu }k^{2}\mu ,k=k,a_{0}=-\frac{2\nu k^{3}\mu^{2}}{\lambda },a_{1}=0,a_{2}=\frac{6\nu k^{3}\mu^{2}}{\lambda },\;b_{1}=0,\;and\;b_{2}=0.$$

Assemble 2’s elements influencing the *coth* functions provide a clear result that may be changed by the time-space coordination, hyperbolic formula, and translation of (4.2.1).


$$U_{2_{3}}=-2+6\frac{1}{{coth(2\sqrt{x}-4\sqrt{t})}^{2}}.$$ (4.2.9)

The results (4.2.9) can be written as follows using the trigonometric formula:

$$U_{2_{4}}=-2+6\{1-{\mathrm{sech}\left(2\sqrt{x}-4\sqrt{t}\right)}^{2}\}.$$ (4.2.10)

## Assemble 3


$$c=4\sqrt{\nu }k^{2}\mu ,k=k,a_{0}=\frac{4\nu k^{3}\mu^{2}}{\lambda },a_{1}=0,a_{2}=\frac{6\nu k^{3}\mu^{2}}{\lambda },\;b_{1}=0,\;and\;b_{2}=\frac{6\nu k^{3}\mu^{2}}{\lambda }.$$

Assemble 3’s elements prompting the *tanh*^2^ and *coth*^2^ functions deliver a clear solution that may be rearranged by applying the time-space coordination, hyperbolic formula, and transformation of (4.2.1).


$$U_{2_{5}}=4+6{tanh(2\sqrt{x}-8\sqrt{t})}^{2}+6{coth(2\sqrt{x}-8\sqrt{t})}^{2}.$$ (4.2.11)

## Assemble 4


$$c=2\sqrt{-\nu }k^{2}\mu ,k=k,a_{0}=-\frac{6\nu k^{3}\mu^{2}}{\lambda },a_{1}=a_{2}=b_{1}=0,\;and\;b_{2}=\frac{6\nu k^{3}\mu^{2}}{\lambda }.$$

Here we take *ν* = −1. The *coth* functions of Assemble 4 propose an explicit solution that can be changed using the time-space coordination, hyperbolic method, and transformation of (4.2.1).


$$U_{2_{6}}=6-6{coth(2\sqrt{x}-4\sqrt{t})}^{2}.$$ (4.2.12)

## Assemble 5


$$c=2\sqrt{-\nu }k^{2}\mu ,k=k,a_{0}=-\frac{6\nu k^{3}\mu^{2}}{\lambda },a_{1}=0,a_{2}=\frac{6\nu k^{3}\mu^{2}}{\lambda },b_{1}=0,\;and\;b_{2}=0.$$

Here we take *ν* = −1. An explicit result, that can be reorganized by using the time-space coordination, hyperbolic method, and use of the transformation of (4.2.1) for assembly 5 are some of the components that affect the tanh functions.


$$U_{2_{7}}=6-6{tanh(2\sqrt{x}-4\sqrt{t})}^{2}.$$ (4.2.13)

## Assemble 6


$$c=4\sqrt{-\nu }k^{2}\mu ,k=k,a_{0}=-\frac{12\nu k^{3}\mu^{2}}{\lambda },a_{1}=0,\;a_{2}=\frac{6\nu k^{3}\mu^{2}}{\lambda },b_{1}=0,\;and\;b_{2}=\frac{6\nu k^{3}\mu^{2}}{\lambda }.$$

Here we take *ν* = −1. The elements of Assemble 6 that stimulus the *tanh* and *coth* functions include an explicit solution that can be reorganized by means of the hyperbolic formulation, time-space coordination, and using the transformation of (4.2.1).


$$U_{2_{8}}=12-6{tanh(2\sqrt{x}-8\sqrt{t})}^{2}-6{coth(2\sqrt{x}-8\sqrt{t})}^{2}.$$ (4.2.14)

It is plausible to conclude that the solution $U_{2_{1}}-U_{2_{8}}$ of general space-time fractional order coupled Boussinesq equation is revolutionary and that this article was the first to discover it, which had previously been unexplored.

## 5 Physical description and visual explanation

Through this segment, we will look at the physical explanations for the well-known solutions of traveling wave for the space-time fractional couple BB and couple Boussinesq equations. In a 3D cartesian coordinate system, the plotline is defined as a coiled or planar surface (a). 3D may be exhibited from all angles with a simple camera turn in a photograph. A contour line is a curve that joins sites where a two-variable function has similar values, as shown in the graph (b). We may determine the thickness of two surfaces represented via a list point plot in (c). Finally, we may determine the direction of a wave by the plot of vector shown in (d) [37]. These plots were visualized by Mathematica. We can more explicitly describe the physical sketch using those four types of pictorial descriptions.

**Fig 1:**
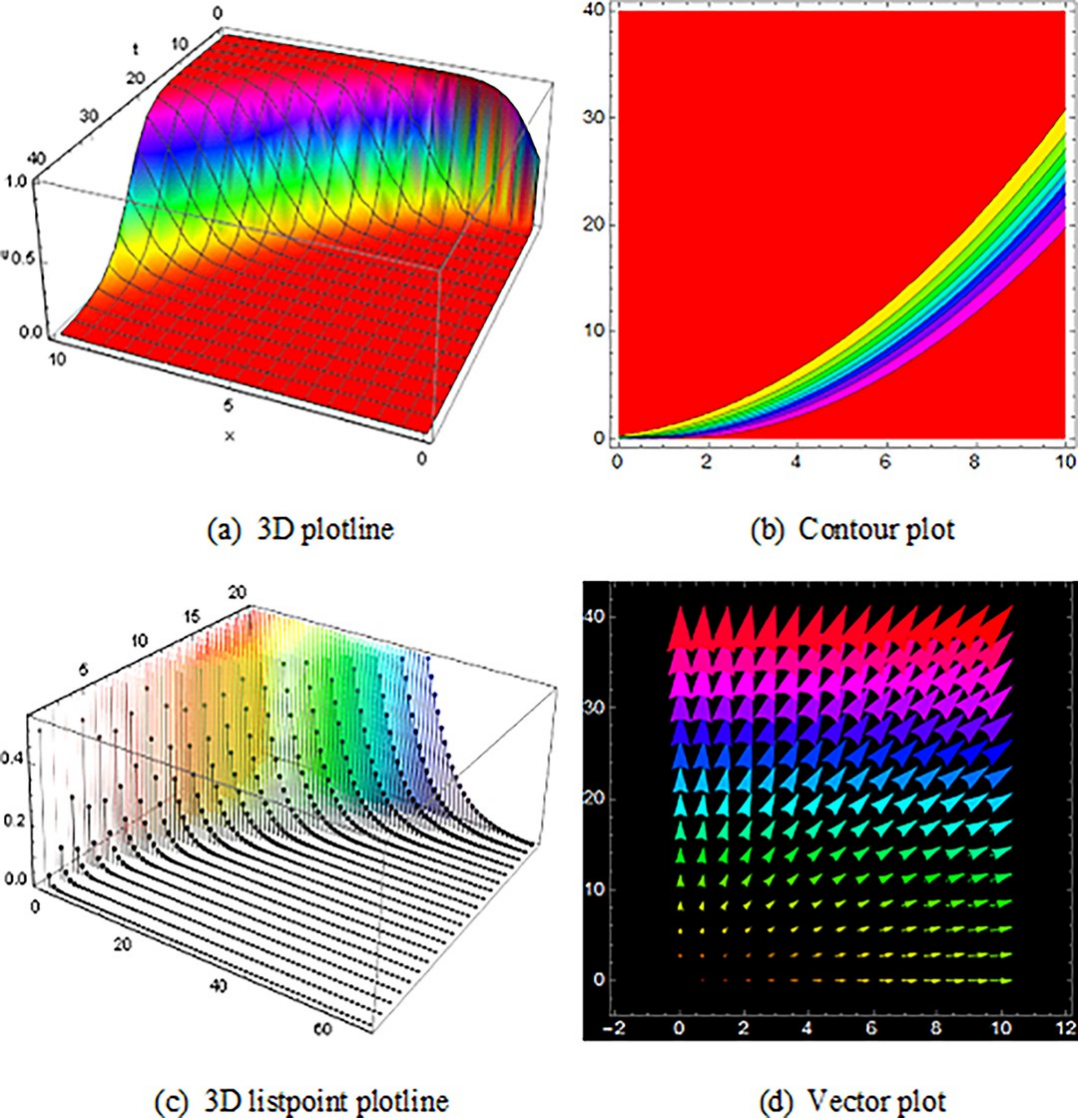
The wave solution of (4.1.7A), Illustration the kink shape presenting the 3D plotline (a), contour plot (b), 3D listpoint plotline (c) and vector plot (d) of $U_{1_{1}}(x,t)$ with interval 0<*x*<10 and 0<*t*<10.

**Fig 2:**
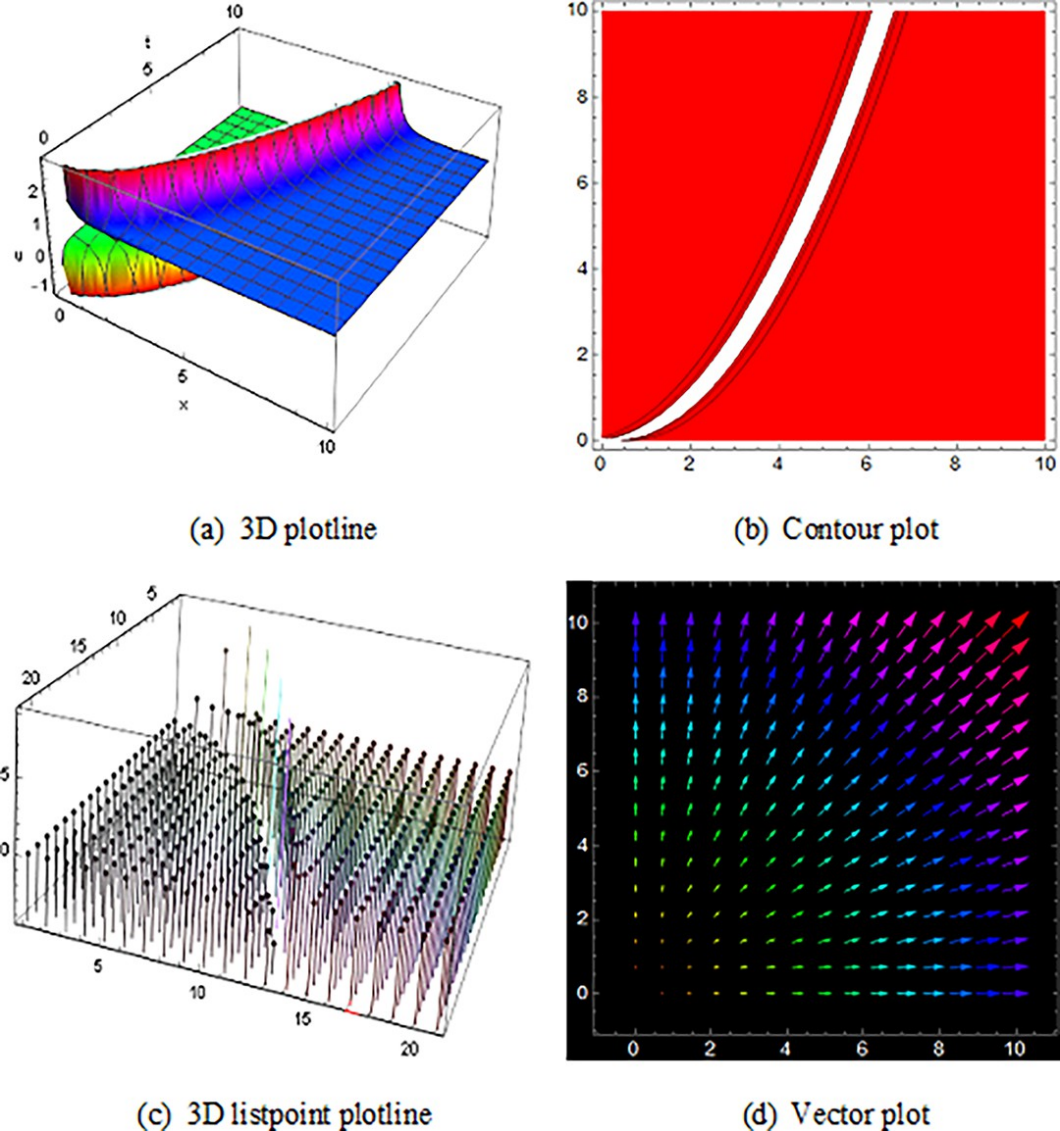
Structure of singular-kink form wave result for (4.1.7A), presenting the 3D plotline (a), contour plot (b), 3D listpoint plotline (c) and vector plot (d) of $U_{1_{6}}(x,t)$ with interval 0<*x*<10 and 0<*t*<10.

The solutions of $U_{1_{1}}(x,t)$ in Fig 1 and $U_{1_{2}}(x,t)$ within the duration 0<*x*<100 *and* 0<*t*<2000 are the result of the space-time fractional coupled BB equation, represented kink shape result of traveling wave that have infinite wings on both sides. The results of $U_{1_{6}}(x,t)$ in (Fig 2) is the singular-kink shape solution of traveling wave and the solutions of $V_{1_{3}}(x,t)$within the duration 0<*x*<10 *and* 0<*t*<10 and $U_{1_{5}}(x,t)$within the duration 0<*x*<10 *and* 0<*t*<10 are also epitomized exactly equivalent result to the space-time fractional order coupled BB equation with infinite wings or infinite tails.

**Fig 3:**
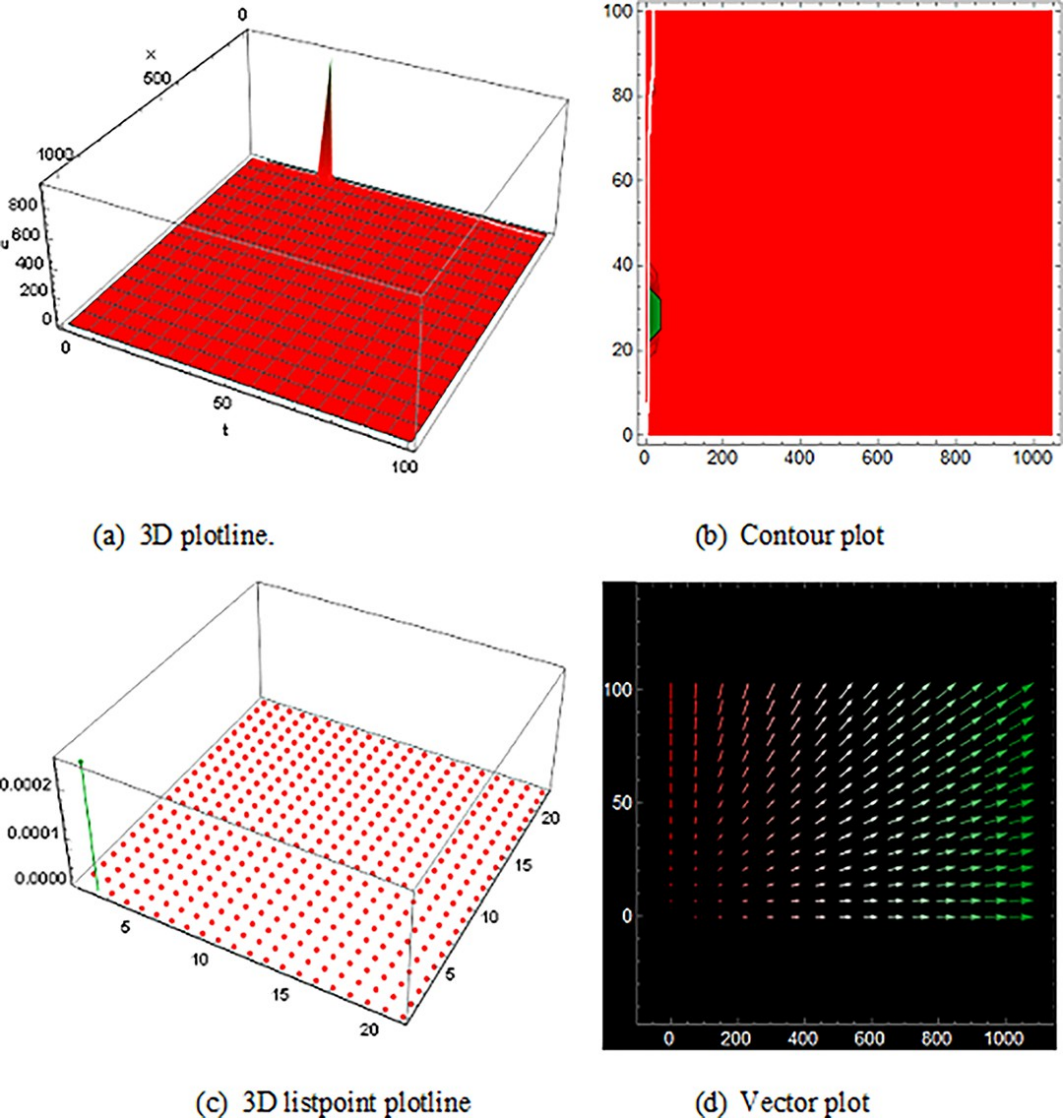
The wave solution (4.1.12B), presenting the single soliton shape and define the 3D plotline (a), contour plot (b), 3D listpoint plotline (c) and vector plot (d) of $V_{1_{6}}$(x, t) with interval 0<*x*<1050 and 0<*t*<100.

The picture of (Fig 3) represents the single soliton wave form solution of $V_{1_{6}}(x,t)$. Singular solitons were the type of solitary wave with a singularity (typically an infinite discontinuity) [38]. Singular solitons are tied to the imaginary center point of a solitary wave. [39]. The solution of $U_{2_{8}}(x,t)$ with the duration 0<*x*<6000 and 0<*t*<2500 are also represented analogous traveling wave solution of the space-time fractional coupled BB equation.

**Fig 4:**
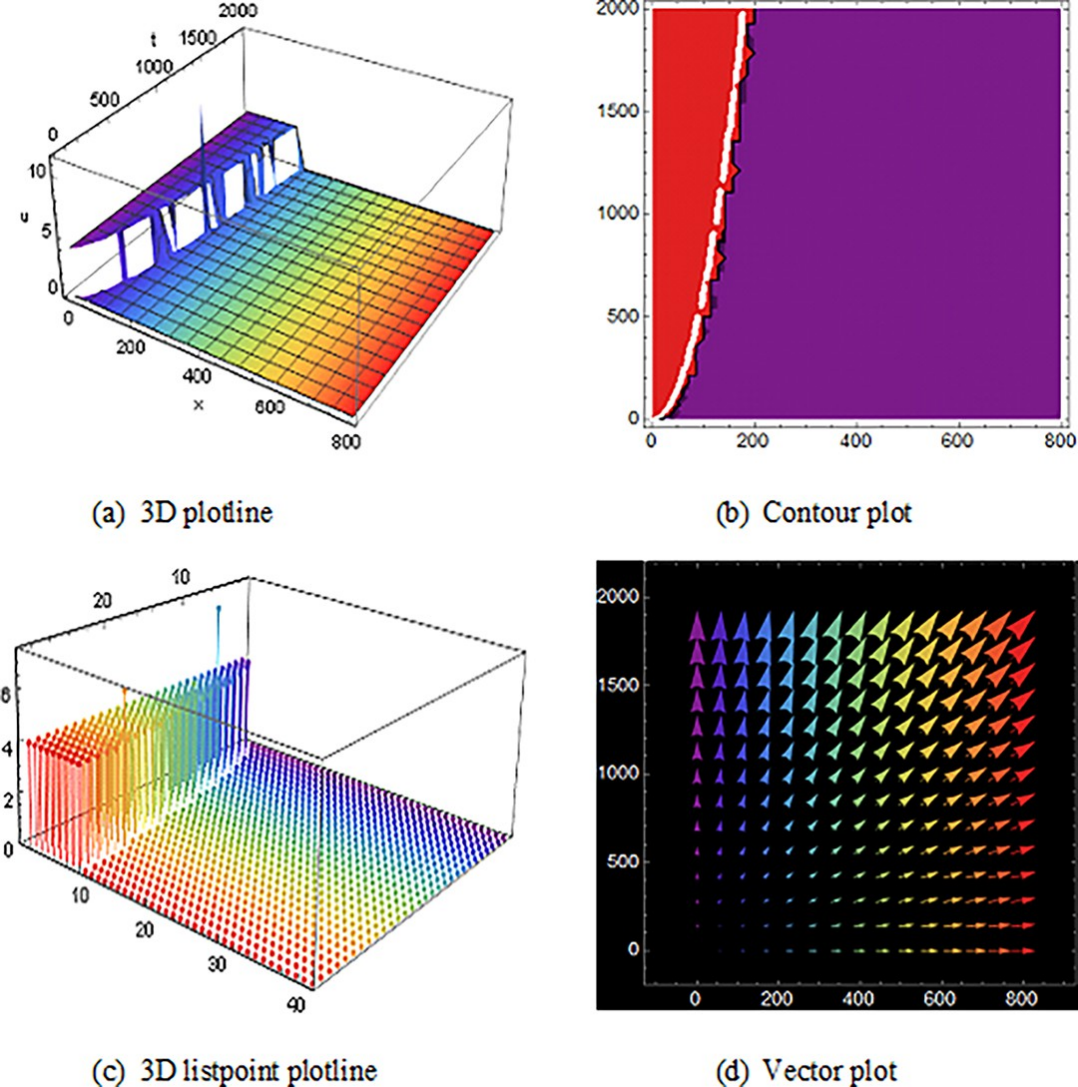
Structure of the multiple soliton form solution of traveling wave (4.1.11B), presenting the 3D plotline (a), contour plot (b), 3D listpoint plotline (c) and vector plot (d) of $V_{1_{5}}$(x, t) with interval 0<*x*<800 and 0<*t*<2000.

**Fig 5:**
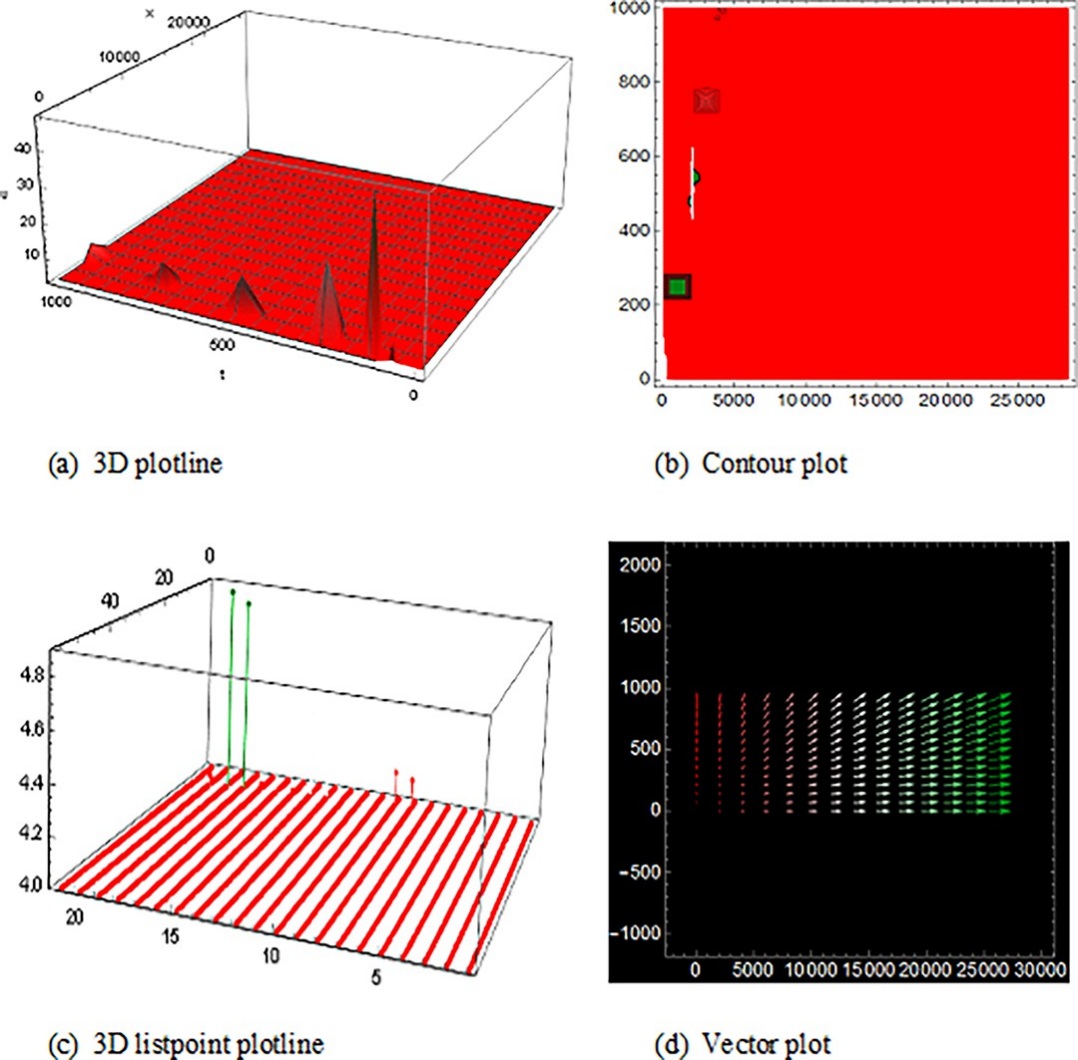
The wave solution (4.2.8), Illustration of the multiple soliton shape that presenting the 3D plotline (a), contour plot (b), three-dimensional listpoint plotline (c) and vector plot (d) of $U_{2_{2}}(x,t)$with interval 0<*x*<28450 and 0<*t*<1000.

In addition, (Figs 4 and 5) and $U_{1_{3}}(x,t)$ with the duration 0<*x*<1250 and 0<*t*<2000 illustrate the numerous solitons using the space-time fractional coupled BB equations.

**Fig 6:**
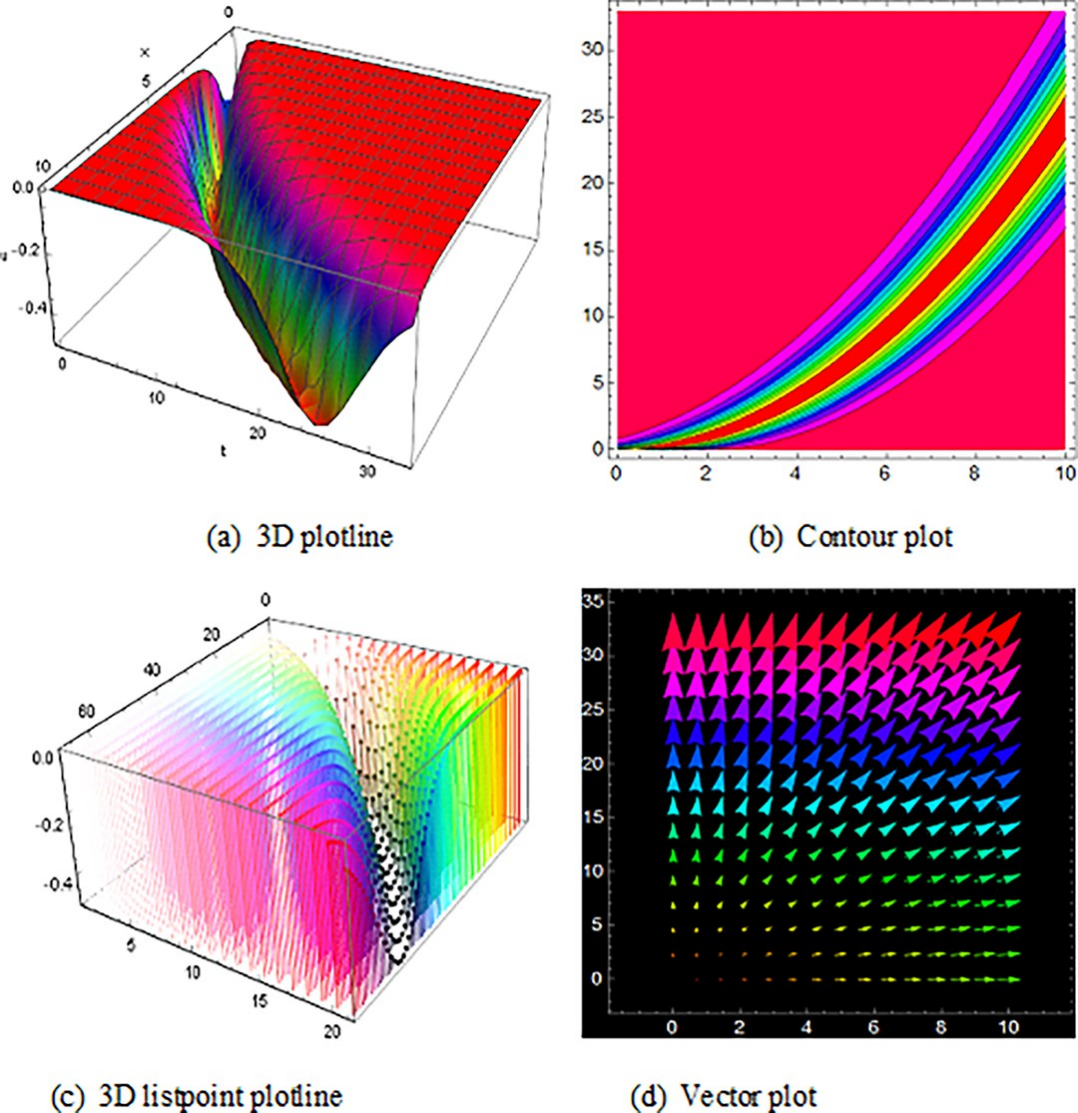
Structure of solution (4.1.7B) is the bell shape wave, presenting the 3D plotline (a), contour plot (b), 3D listpoint plotline (c) and vector plot (d) of $V_{1_{1}}$(x, t) with interval 0<*x*<10 and 0<*t*<33.

**Fig 7:**
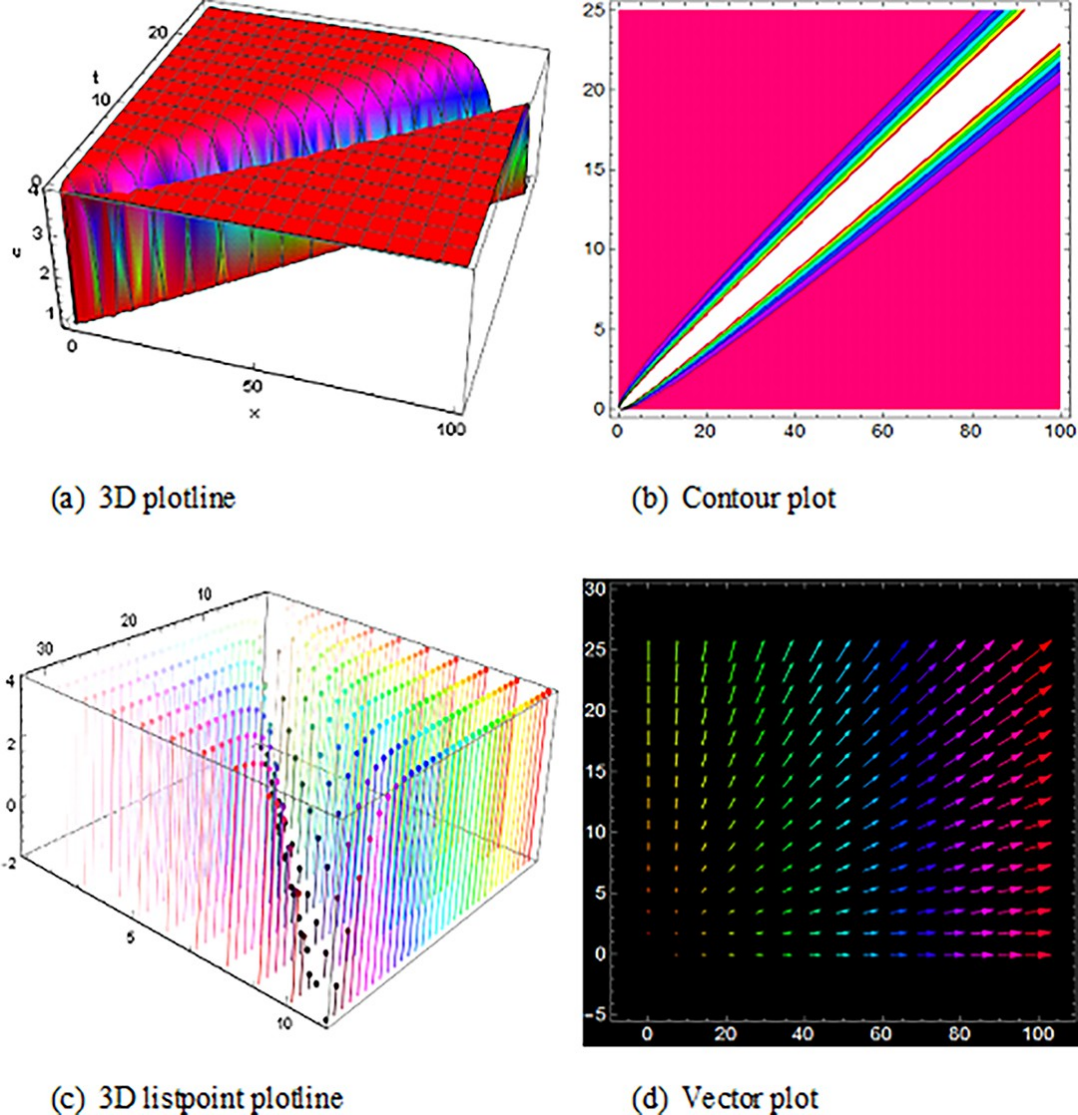
The diagram shows the singular bell form wave result (4.2.10), presenting the 3D plotline (a), contour plot (b), 3D listpoint plotline (c) and vector plot (d) of $U_{2_{4}}$(x, t) with interval 0<*x*<100 and 0<*t*<25.

The profile of the solutions of $V_{1_{1}}(x,t)$is show off (Fig 6), which is the solutions of bell shape compaction would be a soliton that is stable and has compact support i.e., does not have an infinite number of wings for the space-time fractional order coupled type BB equation. Singular bell shape solution found by the space-time fractional order coupled type Boussinesq equations of $U_{2_{4}}(x,t)$in (Fig 7) also $U_{2_{1}}(x,t)$ within the duration 0<*x*<90 *and* 0<*t*<20,$U_{2_{5}}(x,t)$ within the duration 0<*x*<100 *and* 0<*t*<10 and $U_{2_{6}}(x,t)$ with the duration 0<*x*<70 *and* 0<*t*<60 are construct a familiar wave shape of familiar equation.

**Fig 8:**
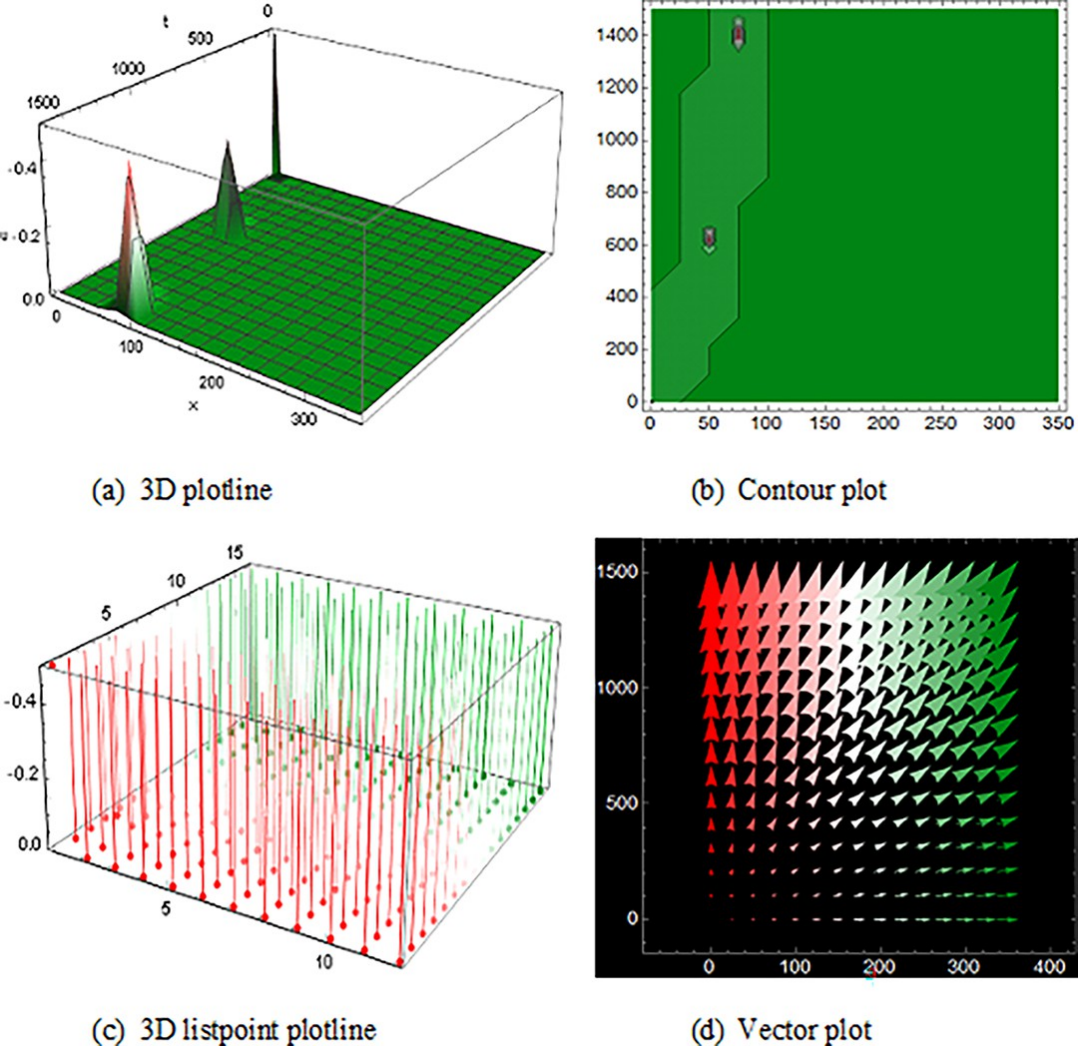
Structure of the triple soliton shape wave result (4.1.8B), presenting the 3D plotline (a), contour plot (b), 3D listpoint plotline (c) and vector plot (d) of $V_{1_{2}}$(x, t) with interval 0<*x*<350 and 0<*t*<1500.

**Fig 9:**
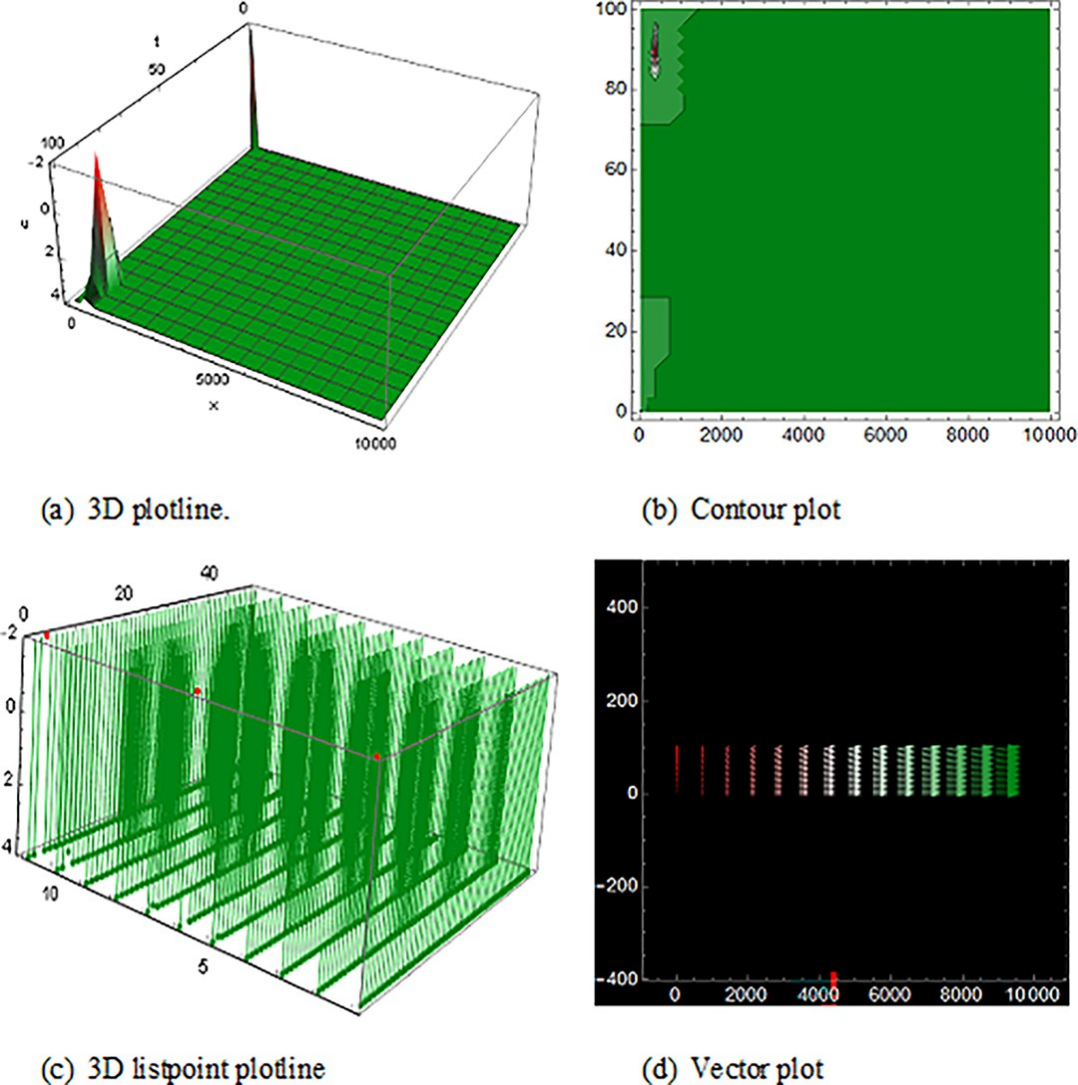
The wave solution (4.2.9), Structured of the double soliton shape presenting the 3D plotline (a), contour plot (b), 3D listpoint plotline (c) and vector plot (d) of $U_{2_{3}}$(x, t) with interval 0<*x*<10000 and 0<*t*<100.

Then, the spike of (Fig 8) is like a triple soliton shape solution of traveling wave for $V_{1_{2}}(x,t)$ of the space-time fractional order coupled type BB equation. Spike of (Fig 9) is like a double soliton shape solution of traveling wave $U_{2_{3}}(x,t)$ also $U_{2_{7}}(x,t)$ with duration 0<*x*<3000 and 0<*t*<6000 construct a similar wave shape traveling wave solution for space-time fractional order coupled type Boussinesq equation.

**Fig 10:**
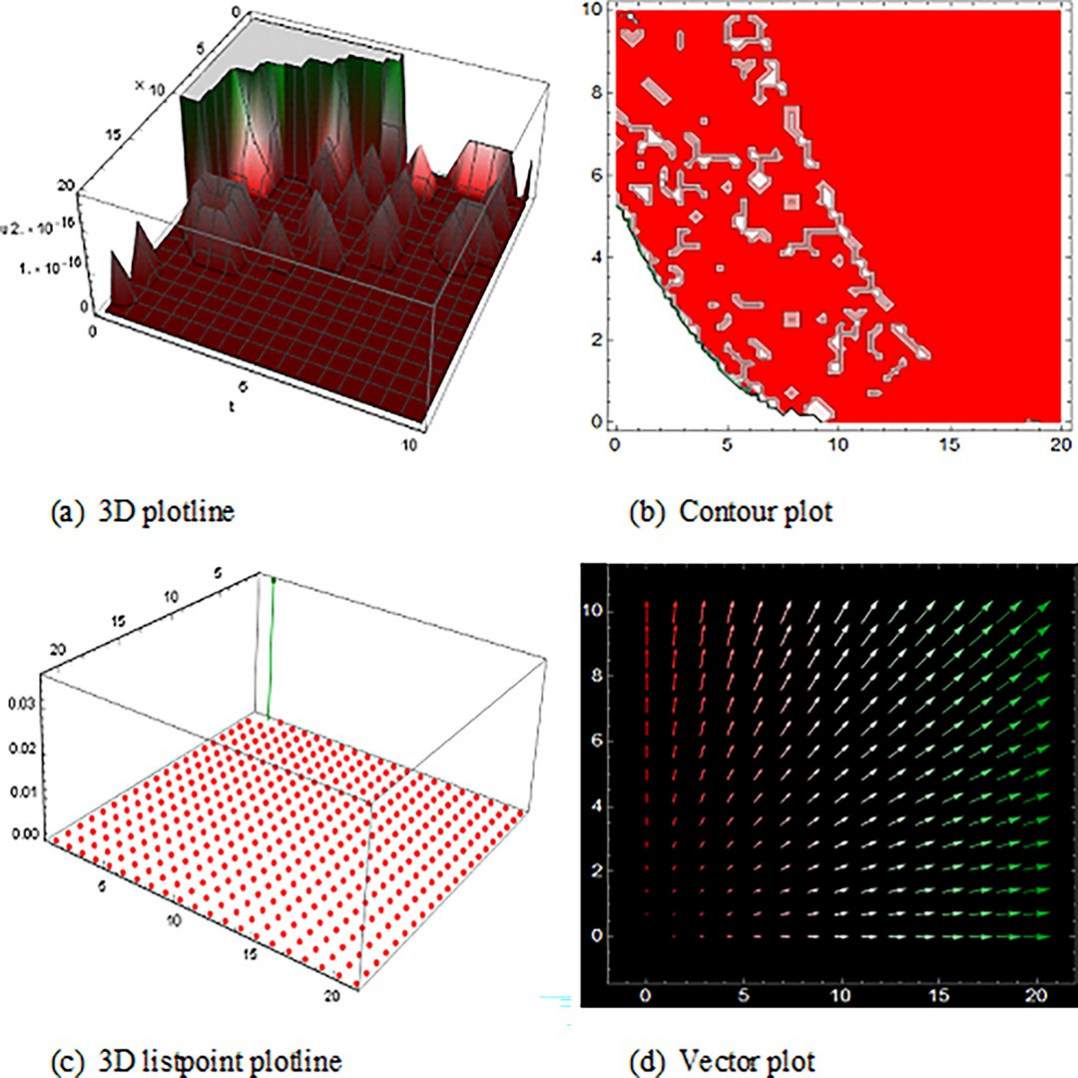
The singular periodic shape wave result of (4.1.10A), presenting the 3D plotline (a), contour plot (b), 3D listpoint plotline (c) and vector plot (d) of $U_{1_{4}}$(x, t) with interval 0<*x*<20 and 0<*t*<10.

**Fig 11:**
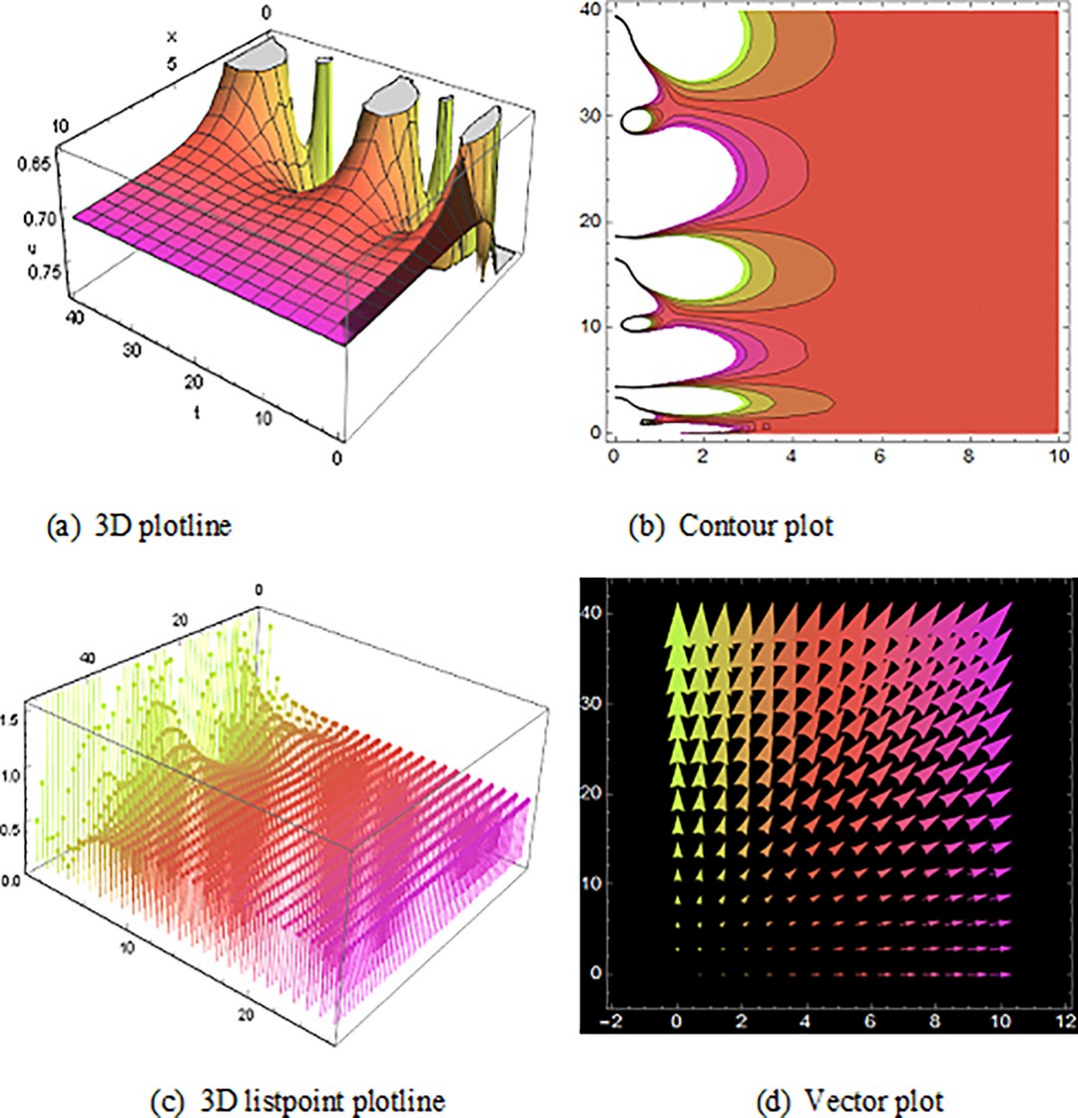
Figure of the imaginary wave result (4.1.13A), presenting the 3D plotline (a), contour plot (b), 3D listpoint plotline (c) and vector plot (d) of $U_{1_{7}}$(x, t) with interval 0<*x*<10 and 0<*t*<40.

**Fig 12:**
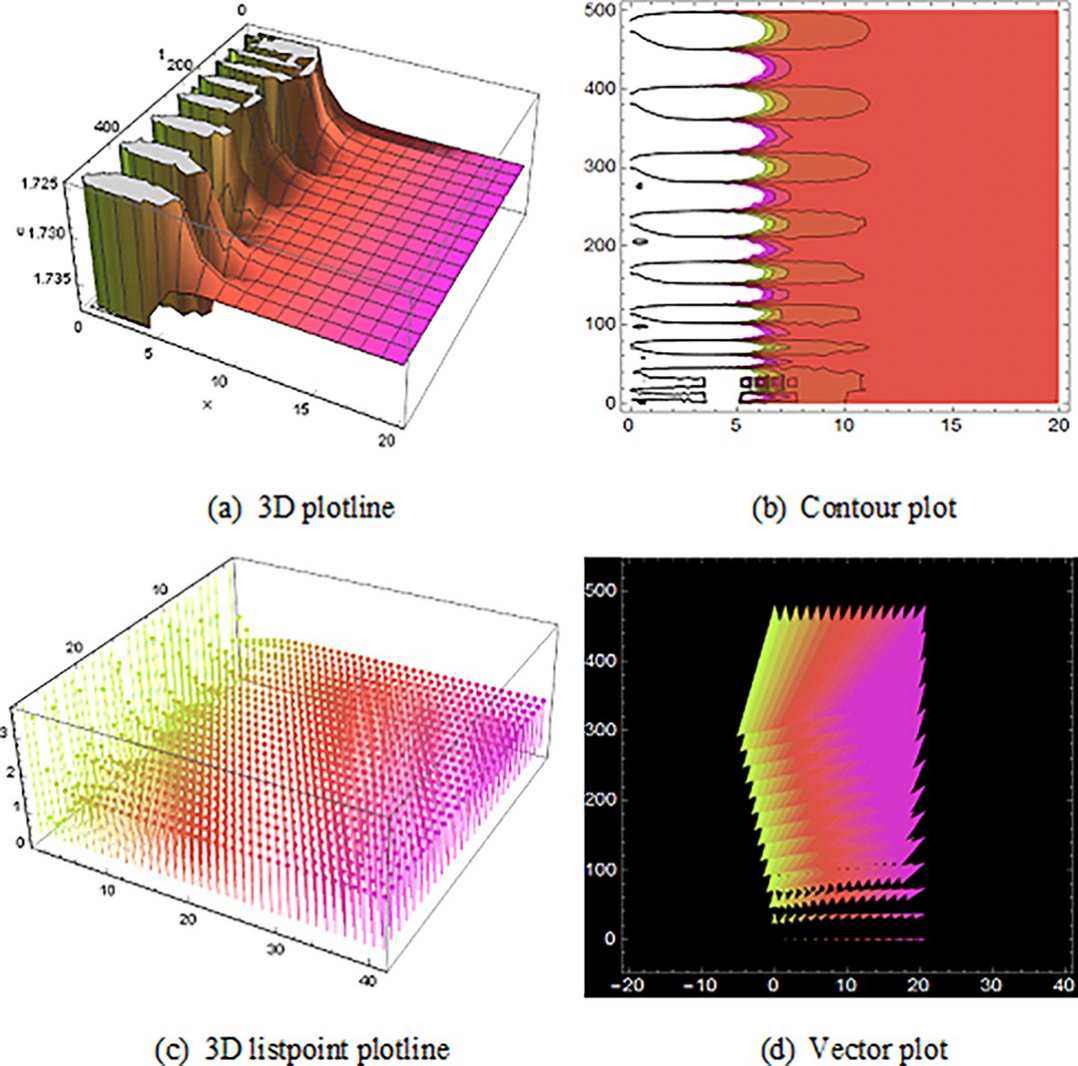
Structure of the imaginary wave solution (4.1.13B), presenting the 3D plotline (a), contour plot (b), 3D listpoint plotline (c) and vector plot (d) of $V_{1_{7}}$(x, t) with interval 0<*x*<20 and 0<*t*<500.

At last, (Fig 10) and $V_{1_{4}}(x,t)$ with the duration 0<*x*<10 and 0<*t*<10 illustrate the periodic shape solution for the space-time fractional coupled BB equation. Finally, (Figs 11 and 12) are the imaginary wave shape of the space-time fractional coupled BB equation.

The graphics for the space-time fractional coupled BB and coupled Boussinesq equation solutions are established here and characterize the different types of pictures which are got by those equations also avoid the similar type wave shape that overlaps to the existing image.

## 6 Conclusion

In this study, the recommended extended tanh-function technique and the conformable derivative definition, we have been able to accomplish and symbolically achieve several types of fresh exact solutions of traveling wave to space-time fractional coupled BB and coupled Boussinesq equations. We discovered some closed form solutions for those mentioned equations, as well as kink-shaped, singular-kink shaped, singular periodic shaped, singular bell-shaped, single soliton formed, bell-shaped, multiple solitons shaped, double soliton shaped, triple soliton shaped, imaginary wave-shaped through various free parameters in the feature of 3D, contour, list point, and vector plots. It is important to keep in mind that the value of the unidentified coefficients evaluate with the use of Maple or Mathematica software. It is important to note that all derived solutions are directly replaced with the original equations to ensure their accuracy.

The achieved solutions are applicable to investigate the transmission of shallow water waves, flow within vertically well-mixed water bodies, water body motion, and many more. Coastal engineers gain advantage highly from a well understanding of their particular theory of nonlinear water wave solutions for generating harbors and coastlines. Accordingly, the different developments of exact traveling wave solutions described in this paper may have a big impact on the investigation of fluid flow and wave motion in the ocean. The adopted method is conformable, trustworthy, direct, and effective; besides it offers a variety of unique physical model solutions to NLPFEEs in engineering, applied mathematics, and mathematical physics. This approach can be used to examine additional nonlinear problems that emerge in theoretical physics, applied mathematics, and other sectors of non-linear sciences.
